# Effectiveness of a Closed-System Transfer Device in Reducing Surface Contamination in a New Antineoplastic Drug-Compounding Unit: A Prospective, Controlled, Parallel Study

**DOI:** 10.1371/journal.pone.0159052

**Published:** 2016-07-08

**Authors:** Nicolas Simon, Michèle Vasseur, Marine Pinturaud, Marion Soichot, Camille Richeval, Luc Humbert, Michèle Lebecque, Ousseini Sidikou, Christine Barthelemy, Pascal Bonnabry, Delphine Allorge, Bertrand Décaudin, Pascal Odou

**Affiliations:** 1 EA 7365 –GRITA—Groupe de Recherche sur les formes Injectables et les Technologies Associées, F-59000, Lille, France; 2 CHU Lille, Institut de Pharmacie, F-59000, Lille, France; 3 Laboratoire de Toxicologie, Hôpital Lariboisière, Assistance Publique-Hôpitaux de Paris, Paris, France; 4 Unité Fonctionnelle de Toxicologie, Pôle de Biologie-Pathologie-Génétique, CHRU Lille, F-59037, Lille, France; 5 Pharmacy, Geneva University Hospitals and School of Pharmaceutical Sciences, University of Geneva, University of Lausanne, Geneva, Switzerland; University of South Alabama Mitchell Cancer Institute, UNITED STATES

## Abstract

**Background:**

The objective of this randomized, prospective and controlled study was to investigate the ability of a closed-system transfer device (CSTD; BD-Phaseal) to reduce the occupational exposure of two isolators to 10 cytotoxic drugs and compare to standard compounding devices.

**Methods and Findings:**

The 6-month study started with the opening of a new compounding unit. Two isolators were set up with 2 workstations each, one to compound with standard devices (needles and spikes) and the other using the Phaseal system. Drugs were alternatively compounded in each isolator. Sampling involved wiping three surfaces (gloves, window, worktop), before and after a cleaning process. Exposure to ten antineoplastic drugs (cyclophosphamide, ifosfamide, dacarbazine, 5-FU, methotrexate, gemcitabine, cytarabine, irinotecan, doxorubicine and ganciclovir) was assessed on wipes by LC-MS/MS analysis. Contamination rates were compared using a Chi^2^ test and drug amounts by a Mann-Whitney test. Significance was defined for p<0.05. Overall contamination was lower in the “Phaseal” isolator than in the “Standard” isolator (12.24% vs. 26.39%; p < 0.0001) although it differed according to drug. Indeed, the contamination rates of gemcitabine were 49.3 and 43.4% (NS) for the Standard and Phaseal isolators, respectively, whereas for ganciclovir, they were 54.2 and 2.8% (p<0.0001). Gemcitabine amounts were 220.6 and 283.6 ng for the Standard and Phaseal isolators (NS), and ganciclovir amounts were 179.9 and 2.4 ng (p<0.0001).

**Conclusion:**

This study confirms that using a CSTD may significantly decrease the chemical contamination of barrier isolators compared to standard devices for some drugs, although it does not eliminate contamination totally.

## Introduction

Since occupational exposure to antineoplastic drugs was first highlighted [1] numerous studies have stated that contamination is common wherever the drugs may be used, whether in hospital or in patients’ homes [2–5]. Professional risks, such as genotoxic effects, or even diminished reproductive health, are associated with the use of these drugs [6,7].

Recommendations have been published and since the early 1980s, professionals [8–10], and institutions like OSHA or the NIOSH have published their own guidelines [11–13]. Other organisms such as the Association of Health-System Pharmacists, the Oncology Nursing Society, the International Society of Oncology Pharmacy Practitioners or the GERPAC-Europharmat workgroup [14–16] have recently put forward their recommendations as well. All concord in advising workers to use collective equipment (vertical laminar air-flow hoods placed in ISO 6 rooms or barrier isolators placed in ISO-7 rooms), to wear personal protective equipment (gloves, masks, gowns) and to use appropriate compounding devices that avoid the use of needles and limit the risk of spillage and leakage.

ISOPP indicates three categories of devices: 1) devices to protect the handler from the vial/ampoule, 2) devices to protect the operator during preparation and 3) devices to protect the administrator during administration of the cytotoxic drug to the patient [17]. Over the last decade, many air venting devices intended to protect the operator during preparation have been marketed: Chemoclave [18,19], Chemoprotect [20], Chemosafe [19], Clave [21–24], Equashield [25,26], Onguard [18,20], Securmix [27], Smart site [20], and Tevadaptor [24,28,29]. While these devices have been shown to help in decreasing contamination by antineoplastic drugs, some of them are classified as closed-system transfer devices (CSTDs). Considering the definition given by the NIOSH, CSTDs mechanically prevent the transfer of environmental contaminants into the system and the escape of hazardous drug or vapor concentrations outside the system. Among them, the Phaseal system (Becton-Dickinson) has been the most studied device in *in vitro* studies or studies in real conditions [30–45]. In the latest studies, Phaseal has been shown to significantly limit environmental contamination by cyclophosphamide by reducing the number of contaminated samples as well as the residual drug amount [42]. However, higher levels of contamination have sometimes been detected after implementation of this device [44].

Most of the assessments of Phaseal effectiveness in real practice conditions have been before/after studies whose major limitation is the inability to distinguish between the impact of the device itself and the quality improvement program provided. To rectify this, we have designed a controlled prospective study in parallel groups comparing Phaseal to standard compounding devices in a new antineoplastic-drug compounding unit.

The aims of this study were to assess the ability of Phaseal to reduce the surface contamination of barrier isolators in real practice conditions and to assess its usability by pharmacy technicians.

## Materials and Methods

### Description of the compounding unit

**Fig 1:**
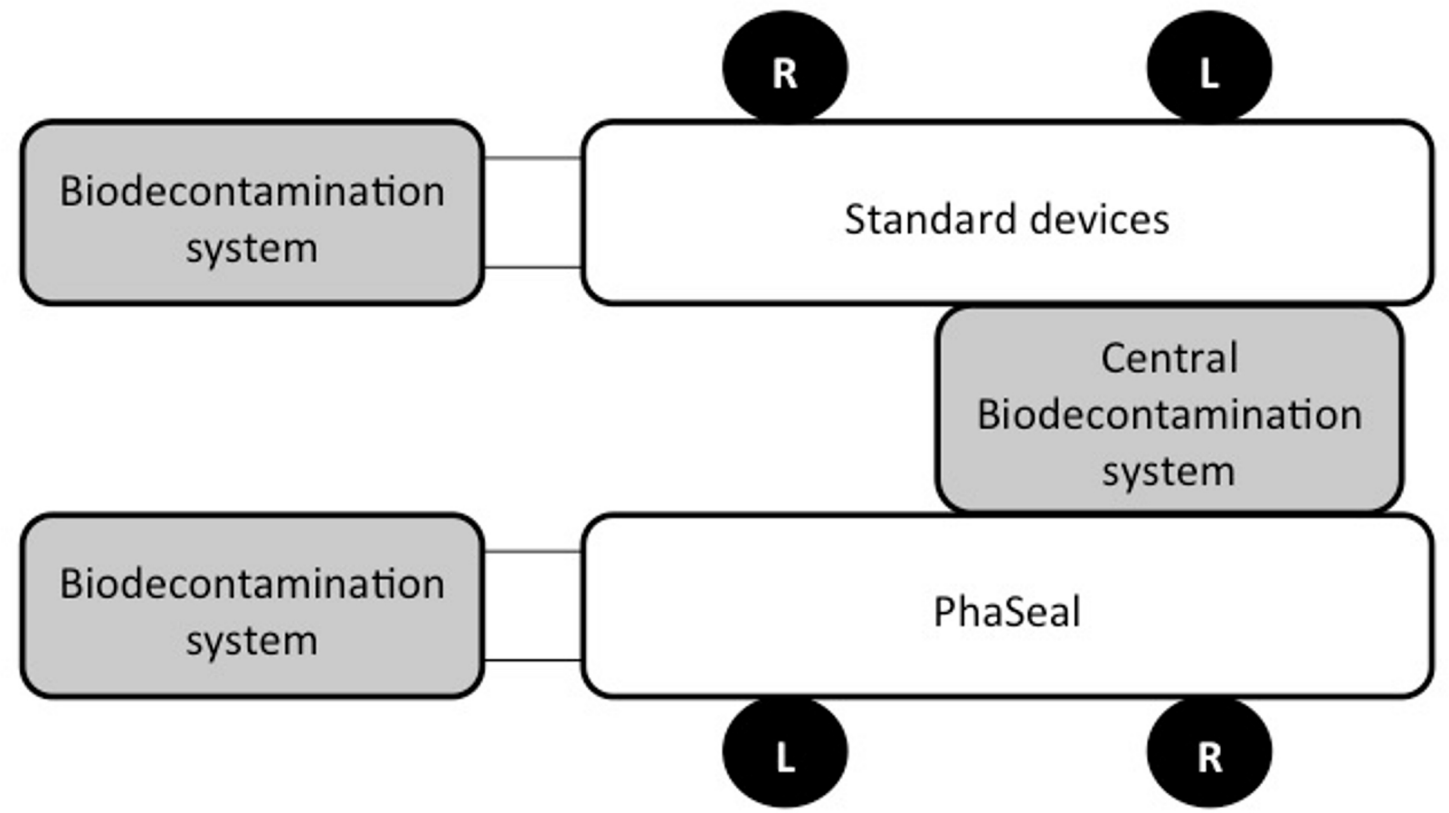
Scheme of the isolators and biodecontamination systems. The two isolators have two workstations each. In one isolator (Standard devices), the preparations are compounded only with spikes and needles. In the other isolator (Phaseal), the compounding process involves only Phaseal devices. Three biodecontamination systems are connected to the isolators. Sterilization is performed with hydrogen peroxide. The central biodecontamination system is an aeraulic barrier. There is no airflow between the two isolators.

This study took place in the new antineoplastic-drug compounding unit located in a university hospital. An average of forty thousand chemotherapy preparations are completed annually by the pharmaceutical team. The unit is staffed by two pharmacists and eight pharmacy technicians. The average work anteriority of the team is 5 years (1–10 years). The new unit is an ISO 7 controlled-atmosphere area equipped with two barrier isolators (Eurobioconcept, Ivry-sur-Seine, France) each with two workstations. These isolators are maintained in depression compared to the environment (-70 Pa) and vented towards the outside. They are connected to three biodecontamination systems (Fig 1), the central one of which is connected to both isolators. This central system is an aeraulic barrier between isolators, as there cannot be any back airflow between two isolators. Hydrogen peroxide (35%, Bioquell, Bonneuil-sur-Marne, France) is used for sterilization and to control microbial contamination and a cleaning process is performed daily in each isolator with a standard quaternary ammonium solution (Anioxispray, Anios, Lille, France). A full cleaning process and sterilization are performed every 14 days unless there is a rupture in sterilisation. Neoprene gloves are changed at the same time.

In the event of a critical incident, activity in the isolator is halted. All protective equipment (gloves, sterile drapes, compresses) is then thrown away and replaced, drug vials are cleaned by compresses and the isolator with an antimicrobial solution.

### Study design

#### Description of groups and compounding process

The study started at the opening of the unit and lasted 6 months. Compounding processes were different in the two isolators. In the first one (S), all preparations were compounded with standard medical devices (needles and vented needle-free devices) BD-Microlance (Becton Dickinson, Le Pont de Claix, France) and Microspikes (BBraun, Boulogne-Billancourt, France). In the second one (P), only BD Phaseal devices (Becton Dickinson, Le Pont de Claix, France) were used. Drug withdrawal from vials was carried out with an adapted Protector (BD-Protector P14; P21; P50) and an Injector (BD-Injector N35) connected to a three-piece syringe (BD-Plastipak, Le Pont de Claix, France). The transfer of drug into the dilution bag or syringe was performed with a Connector (BD-Connector C45) connected to a specific extension set (Cyto-Ad set, Codan, Vallauris, France). The pharmacy technicians were trained by the BD-training team to use Phaseal devices before the study began and were relayed by hospital pharmacists. The former had in fact been using the system in the old compounding unit for several weeks before the study started.

The same work-time was devoted to each isolator. Each pharmacy technician was affected to one or the other group. The compounding process with needles and spikes was identical for each pharmacy technician. The quality of this compounding process was the same in relation to their experience.

#### Drug distribution between the two groups

Ten drugs were monitored during the study: cyclophosphamide, cytarabine, dacarbazine, doxorubicin, 5-fluorouracil, ganciclovir, gemcitabine, ifosfamide, irinotecan and methotrexate. Two groups of five drugs were constituted and compounded alternatively on odd and even days in each isolator. Some exceptions were made to ease the workload as this study was performed in routine practice. On the one hand, drugs for intrathecal administration (e.g. cytarabine, methotrexate), monoclonal antibodies, experimental drugs, syringes for chemoembolization and drugs supplied in very small vials (e.g. vincristine) were always compounded with standard devices. In fact, drugs compounded for intrathecal administration (e.g. cytarabine and methotrexate) were chosen not to be prepared with BD-Phaseal so as to avoid any technical risk in administration and because of dead-space volume in the case of low-volume prescription. Monoclonal antibodies were prepared with standard compounding devices because of the risk of generating foam during compounding. Experimental drugs were always compounded in the Standard isolator for administrative reasons and very small vials are not convenient with BD-Phaseal because the volume of solution is very low and difficult to retrieve totally with this device. Finally, syringes prepared for chemoembolization (with doxorubicine) were compounded only in the Standard group.

On the other hand, some drugs (bendamustine, busulfan, cisplatine, carboplatine, etoposide, fludarabine, idarubicine, pemetrexed, paclitaxel, vinorelbine) were always compounded with BD-Phaseal to balance the workload between the two isolators. Finally, less frequently compounded drugs (amsacrine, cidofovir, cladribine, clofarabine, daunorubicine, epirubicine, mitoxantrone, raltitrexed, streptozocine, thiotepa, topotecan and vindesine) were prepared in either isolator, depending on the activity of the day. The pharmacists determined drug distribution after their pharmaceutical analysis of prescriptions.

#### Sampling procedure

Samples consisted in surface wiping with 5 x 5 cm compresses (ref. 22104KL1, Tetramedical, Annonay, France) humidified with 100 μL of water for injection (Mouvaux, Tourcoing, France). The sampling method was developed and validated in the framework of this study: it concerned three surfaces: gloves, inner surface of window, and worktop. Samples were wiped at each workstation before and after the daily cleaning process. For both window and worktop, a 10 cm square was wiped (surface of 100 cm^2^). The sampling was performed at the same time on each sampling day, at the end of the morning compounding process (12 p.m).

At the end of the sampling process (when the surface was dry), the compress was placed inside a 10 mL sterile tube (Falcon, France). Sampling took place on 24 days during the study period: on day 1, then daily for the two first weeks, weekly until month 3, and monthly until month 6. Blank samples were established before surface sampling and consisted of placing a compress in a Falcon tube inside the isolators. Samples were stored at -20°C until dosing. According to a previously published study, analysis must take place within 14 days after sampling due to drug instability [46].

#### Dosing assay

Contamination by antineoplastic drugs was measured using an LC-MSMS method (Xevo TQD, Waters, Guyancourt, France). After placing 75 μL of internal standard solution (methylclonazepam) on compresses, drugs were extracted with 2 mL of 0.1% formic acid in methanol. After 20 min incubation, the whole was centrifuged for 10 min at 4500 rpm. After retrieving the wipe, solvent was evaporated at 40°C under nitrogen stream. The dry residual was dissolved by 100 μL of 0.2% formic acid in acetonitrile. 7.5 μL was then injected into the system and eluted on a stationary phase Acquity UPLC BEH (1.7 μm, 2.1x100 mm). Drug separation took 4 min with a gradient elution using a mobile phase composed of ammonium formate buffer 5 mM, 0.1% formic acid in water/0.1% formic acid in acetonitrile. The method was validated according to the technical guidelines provided by the French National Accreditation organism (COFRAC) for method validation in medical biology [47].

The assay characteristics are summarized in Table 1.

**Table 1:** Assay characteristics for the ten tested drugs.

	Desorption yield (%)	LOD (ng)	LOQ (ng)
**Cyclophosphamide**	106%	1	1
**Cytarabine**	65%	1	10
**Dacarbazine**	105%	1	10
**Doxorubicin**	108%	1	10
**Fluorouracil**	65%	1	10
**Ganciclovir**	74%	1	10
**Gemcitabine**	94%	1	1
**Ifosfamide**	98%	1	1
**Irinotecan**	100%	1	1
**Methotrexate**	97%	1	10

LOD: Limit Of Detection, LOQ: Limit Of Quantification

### Main parameters measured

#### Estimation of occupational exposure

A sample was considered contaminated if at least one out of the 10 drugs was retrieved on the sample (trace or quantifiable drug). Samples were considered non-contaminated if drug amounts were under 1 ng. Traces were considered when drugs were > 1 ng but < LLOQ. For all contaminated samples with a drug amount > LLOQ, contamination was measured.

The mean compounded dose of each drug (in mg) was calculated by taking into account all the preparations compounded during the study period. The contamination rate (CR, in %) was calculated by dividing the number of contaminated samples (n) by the number of measured samples (N). The mean contamination amount (in ng) was calculated per sampling day. For gloves, contamination is expressed as ng/glove. For both worktop and window, it is expressed as ng/cm^2^.

All critical incidents were recorded throughout the study. A critical incident was defined as any contamination which could radically increase the risk of occupational exposure to drugs, such as visible leaks or spill, vial breakage or any problem occurring during the compounding process and suspected of increasing occupational exposure.

To avoid any evaluation bias, the measurement of occupational exposure was carried out in blind conditions. The results were interpreted after the last sampling day.

#### Estimation of the usability of devices by pharmacy technicians

An 11-item form was distributed each month (M1 to M6) to assess encumbrance, ergonomics, impression of safety and to establish a comparison with the standard compounding process.

Ergonomics was assessed via 7 items to describe each step of the compounding process: connection of protector to vials; connection of injector to syringes; connection of injector to protector; injection of liquid into vials; sampling of solution from vials; injection of liquid into containers; sampling of liquids from infusion bags. The other four items were: encumbrance, impression of safety during handling, comparison to the reference method and a global appreciation of the handling process with BD-Phaseal.

Each item was assessed according to a 5-point Likert’s scale: very bad, bad, medium, good, very good.

### Statistical comparisons

The mean compounded doses were compared with a Student’s t test. Contamination rates were compared according to a *Chi*^*2*^ test. For small samples, appropriate tests (*Chi*^*2*^ with Yates’ correction or Fisher’s exact test) were used. Contamination amounts were compared with a non-parametric Mann-Whitney test because of sample number (< 30 samples). The significance value of these tests was defined at 0.05.

## Results

### Study description

The study lasted from April 9^th^ to October 8^th^ 2014. 20,097 chemotherapy doses were prepared during this period. More preparations (n = 12,554) were completed with standard compounding devices, due to a high number of experimental drugs and intrathecal drugs required. At the end of the study, a total of 686 surface samples were measured. All blank samples (before starting the study and for each sampling day) were contamination-free. The delay (m±sd) between sampling and dosing days was 5±3 days, always less than 14 days.

Comparison of the mean compounded doses between both isolators revealed no significant difference, except for cytarabine and methotrexate (Table 2) with significantly higher doses in the Phaseal isolator.

**Table 2:** Compounding activity for the ten drugs studied throughout the study. Data correspond to the number of preparations (N) and prepared doses for each of the 10 studied drugs. Doses are expressed in mg and are presented as mean±standard deviation.

Drugs	Standard		Phaseal		P
	N	Doses	N	Doses	
**Cyclophosphamide**	428	1320±905	453	1237±857	0.163^‡^
**Cytarabine**	957*	1040±1807	642	1564±1872	< 0.0001^‡^
**Dacarbazine**	90	776±434	90	751±414	0.711^‡^
**Doxorubicin**	393**	71±24	229	64±31	0.062^‡^
**Fluorouracil**	1003	2335±1767	1067	2276±1735	0.442^‡^
**Ganciclovir**	960	251±114	524	262±126	0.118^‡^
**Gemcitabine**	254	1728±390	210	1633±323	0.245^‡^
**Ifosfamide**	58	3184±1317	54	3426±1474	0.361^‡^
**Irinotecan**	174	291±62	175	295±64	0.591^‡^
**Methotrexate**	662***	612±1759	251	1782±2736	< 0.0001^‡^

* with 332 syringes for intrathecal administration

** with 41 syringes for chemoembolization and 8 bags for one clinical trial

*** with 183 syringes for intrathecal administration

^‡^ Comparison of compounded doses between Standard and Phaseal groups by the Student’s t test

As for incidents, no vial breakage occurred in either isolator during the study period. No other critical incident occurred with the standard devices whereas one was recorded for 0.7% of preparations compounded with Phaseal. The types of incidents, number of occurrences, drugs concerned and days of occurrence are listed in Table 3. Among the recorded incidents, 4 were due to inadequate knowledge (methotrexate leakages), and 11 were due to procedural errors.

**Table 3:** Critical incidents recorded during the study with Phaseal devices during the compounding of tested drugs.

Incident types	Number of Occurrences	Drugs	Days of occurrence
**Leaks**	4	Methotrexate	27, 34, 48, 114
**Injector disconnection**	3	Methotrexate, cyclophosphamide, fluorouracil	6, 7, 114
**Syringe disconnection**	2	Methotrexate, cytarabine	73, 119
**Connector disconnection**	1	Fluorouracil	14
**Expansion tank trouble**	3	Cyclophosphamide, fluorouracil, gemcitabine	1, 15, 119
**Injector breakage**	1	Gemcitabine	3
**Needle out of injector**	1	Methotrexate	114

### Contamination description

#### Comparison of contamination between Standard and Phaseal devices before cleaning process

Contamination was detected on all tested surfaces from the first to the last study day. Among the 10 tested drugs, some of them (e.g. dacarbazine, doxorubicin and methotrexate) were never retrieved or only traces were sometimes detected. Irinotecan was quantified only once (143.8 ng) on the window of the isolator with a standard device. Dacarbazine was quantified once (82 ng) on the worktop with BD Phaseal.

**Fig 2:**
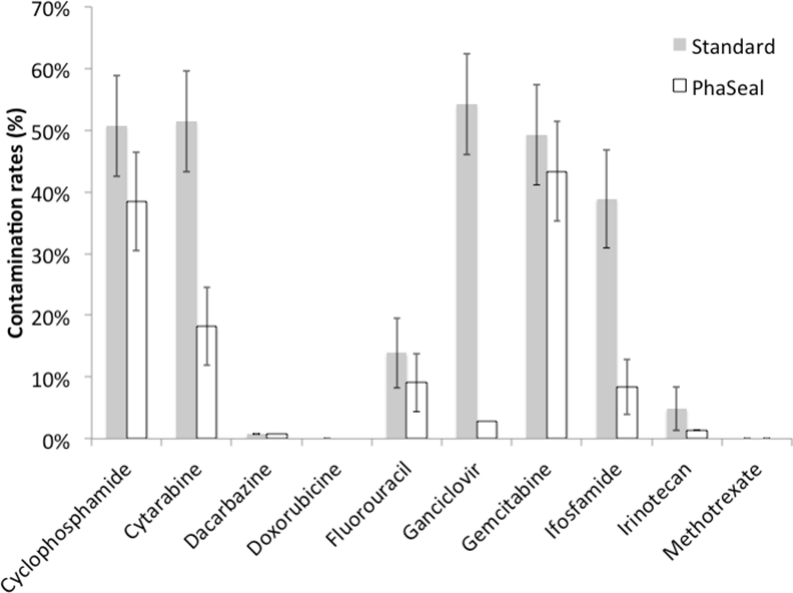
Antineoplastic drug contamination rate of the barrier isolators before cleaning procedure. Histograms represent the proportion of positive samples including traces. Error bars represent the confidence intervals.

As depicted on Fig 2, the contamination rates (CR) before cleaning procedure were systematically lower in the Phaseal group. Specifically, the difference in contamination rate was -12.3%, -33.2%, -4.8%, -51.4%, -5.6%, -30.5% and -3.5% for cyclophosphamide, cytarabine, 5-fluorouracil, ganciclovir, gemcitabine, ifosfamide and irinotecan, respectively.

The contamination rate was significantly lower in the Phaseal isolator whatever the localization. For each isolator, gloves were the most contaminated area in both Phaseal and Standard groups (p < 0.001). Indeed, the percentages of contaminated samples were 33.1%, 24.2%, and 21.9% for gloves, window and worktop, respectively with the standard devices. The trend was similar for Phaseal devices: 16.0%, 10.4%, and 10.0% for gloves, worktop and window, respectively.

Gemcitabine, cyclophosphamide and ifosfamide were the three main contaminating drugs. In the isolator with standard devices, ganciclovir was very frequently quantified on gloves whereas it was less frequently detected on worktop and on inner window surface (Tables 4, 5 and 6).

**Table 4:** Contamination measured for gloves before cleaning process. Results for contamination rates are presented as number of positive samples/number of measured samples (n/N) and in %. Drug amounts are expressed in ng/glove. Drugs are classified according to the frequency of positive samples in the Standard group.

		Standard	Phaseal	P
**Ganciclovir**	CR	38/48 (79.2%)	2/48 (4.2%)	< 0.0001**
	Min	5.7	5.7	-
	Med	19.1	5.7	
	Max	327.7	5.7	
**Cytarabine**	CR	36/48 (75.0%)	16/48 (33.3%)	< 0.0001*
	Min	8.4	8.0	-
	Med	15.3	14.5	
	Max	149.1	226.9	
**Gemcitabine**	CR	30/48 (62.5%)	25/48 (52.1%)	0.302*
	Min	0.65	0.6	-
	Med	16.5	7.1	
	Max	519.9	556.8	
**Cyclophosphamide**	CR	19/48 (39.6%)	16/48 (33.3%)	0.525*
	Min	0.55	0.55	-
	Med	1.9	6.1	
	Max	271.6	88.1	
**Ifosfamide**	CR	18/48 (37.5%)	6/48 (12.5%)	0.005*
	Min	0.55	0.55	-
	Med	2.2	1.2	
	Max	55.7	7.4	
**5-fluorouracil**	CR	14/48 (29.2%)	10/48 (20.8%)	0.346*
	Min	7.5	7.3	-
	Med	23.5	30.3	
	Max	131.6	84.6	
**Irinotecan**	CR	3/48 (6.3%)	1/48 (2.1%)	0.617**
	Min	< LOD	< LOD	-
	Med	< LOD	< LOD	
	Max	< LOD	< LOD	
**Dacarbazine**	CR	1/48 (2.1%)	1/48 (2.1%)	1.00**
	Min	< LOD	< LOD	-
	Med	< LOD	< LOD	
	Max	< LOD	< LOD	

* Comparison of contamination rates between both Standard and Phaseal groups with a Chi^2^ test

** Comparison of contamination rates between both Standard and Phaseal groups with a Fisher’s exact test

**Table 5:** Contamination measured for worktop before cleaning process. Results for contamination rates are presented as number of positive samples/number of measured samples (n/N) and in %. Drug amounts are expressed in ng/cm^2^. Drugs are classified according to the frequency of positive samples in the Standard group.

		Standard	Phaseal	P
**Cyclophosphamide**	CR	25/48 (52.1%)	22/47 (46.8%)	0.607*
	Min	0.018	0.01	-
	Med	0.171	0.03	
	Max	68.45	0.49	
**Gemcitabine**	CR	21/48 (43.8%)	18/47 (38.2%)	0.589*
	Min	0.017	0.025	-
	Med	0.173	0.264	
	Max	4.03	8.93	
**Ifosfamide**	CR	20/48 (41.7%)	4/47 (8.5%)	< 0.001*
	Min	0.011	0.012	-
	Med	0.050	0.046	
	Max	1.28	3.55	
**Ganciclovir**	CR	19/48 (39.6%)	1/47 (2.1%)	< 0.0001**
	Min	0.135	< LOD	-
	Med	0.222	< LOD	
	Max	1.86	< LOD	
**Cytarabine**	CR	17/48 (35.4%)	4/47 (8.5%)	0.004*
	Min	0.236	0.175	-
	Med	0.788	0.175	
	Max	2.85	0.175	
**5-fluorouracil**	CR	3/48 (6.3%)	1/47 (2.1%)	0.617**
	Min	0.258	0.885	-
	Med	2.08	0.885	
	Max	3.91	0.885	

* Comparison of contamination rates between both Standard and Phaseal groups with a Chi^2^ test

** Comparison of contamination rates between both Standard and Phaseal groups with a Fisher’s exact test

**Table 6:** Contamination measured for window before cleaning process. Results for contamination rates are presented as number of positive samples/number of measured samples (n/N) and in %. Drug amounts are expressed in ng/cm^2^. Drugs are classified according to the frequency of positive samples in the Standard group.

		Standard	Phaseal	P
**Cyclophosphamide**	CR	29/48 (60.4%)	17/48 (35.4%)	0.014*
	Min	0.01	0.012	-
	Med	0.08	0.051	
	Max	43.2	23.76	
**Ganciclovir**	CR	21/48 (43.8%)	1/48 (2.1%)	< 0.0001**
	Min	0.116	0.350	-
	Med	0.263	0.350	
	Max	2.62	0.350	
**Cytarabine**	CR	21/48 (43.8%)	6/48 (12.5%)	< 0.001*
	Min	0.103	0.125	-
	Med	0.149	0.167	
	Max	1.13	0.720	
**Gemcitabine**	CR	20/48 (41.7%)	19/48 (39.6%)	0.835*
	Min	0.013	0.017	-
	Med	0.052	0.097	
	Max	0.788	4.40	
**Ifosfamide**	CR	18/48 (37.5%)	2/48 (4.2%)	< 0.0001**
	Min	0.012	0.012	-
	Med	0.130	0.162	
	Max	0.763	0.311	
**Irinotecan**	CR	4/48 (8.3%)	1/48 (2.1%)	0.362**
	Min	1.43	< LOD	-
	Med	1.43	< LOD	
	Max	1.43	< LOD	
**5-fluorouracil**	CR	3/48 (6.3%)	2/48 (4.2%)	1.00**
	Min	2.58	0.258	-
	Med	3.60	0.353	
	Max	4.62	0.449	

* Comparison of contamination rates between both Standard and Phaseal groups with a Chi^2^ test

** Comparison of contamination rates between both Standard and Phaseal groups with a Fisher’s exact test

The use of BD-Phaseal limited contamination for most drugs. For gloves, the use of BD-Phaseal significantly reduced contamination for 3 out of 8 drugs. For the worktop, contamination was significantly decreased for 3 out of 6 drugs, whereas for the inner face of the window, the use of a CSTD significantly reduced contamination for 4 out of 7 drugs. Only for ganciclovir and ifosfamide were contamination rates significantly lower for all three tested surfaces. They were also significantly lower on gloves for ganciclovir and on the inner face of the window for cyclophosphamide.

When data were compared independently of localization, contamination amounts were found to be significantly lower for cyclophosphamide, ifosfamide and ganciclovir whereas no significant difference was apparent for either gemcitabine or fluorouracil. For cytarabine, the results observed were close to significance, but not statistically different (Tables 7 and 8).

**Table 7:** Comparison of contamination rates (in %) and contamination amounts (in ng) between Standard and Phaseal devices according to drug. Contamination rates are computed taking traces into account. Contamination amounts correspond to the cumulative amount per sampling day.

	Gemcitabine			Fluorouracil				Cytarabine		
	Standard	Phaseal	p	Standard	Phaseal	P	Standard	Phaseal	p
**Contamination rate–n/N (%)**	71/144 (49.3%)	62/143 (43.4%)	0.312*	20/144 (13.9%)	13/143 (9.1%)	0.203*	74/144 (51.4%)	26/143 (18.2%)	<0.001*
**Drug amount before cleaning (ng)**									
Min	0.0	0.0	0.493^‡^	0.0	0.0	0.190^‡^	0.0	0.0	0.090^‡^
Median	63.7	22.9		9.9	0.0		17.7	0.0	
Max	1275.1	2777.6		743.6	194.9		460.8	482.1	
**Drug amount after cleaning (ng)**									
Min	0.0	0.0	0.811^‡^	0.0	0.0	0.591	0.0	0.0	0.058^‡^
Median	20.3	16.5		0.0	0.0		0.0	0.0	
Max	768.6	2124.9		4374.3	114.6		319.1	676.0	

* Comparison of contamination rates between both Standard and Phaseal groups with a Chi^2^ test

^‡^ Comparison of contamination amounts between both Standard and Phaseal groups a non-parametric Mann-Whitney test

**Table 8:** Comparison of contamination rates (in %) and contamination amounts (in ng) between Standard and Phaseal devices according to drug. Contamination rates are computed taking traces into account. Contamination amounts correspond to the cumulative amount per sampling day.

	Cyclophosphamide			Ifosfamide			Ganciclovir		
	Standard	Phaseal	p	Standard	Phaseal	p	Standard	Phaseal	p
**Contamination rate–n/N (%)**	73/144 (50.7%)	55/143 (38.5%)	0.037*	56/144 (38.9%)	12/143 (8.4%)	< 0.0001*	78/144 (54.2%)	1/143 (0.7%)	< 0.0001**
**Drug amount before cleaning (ng)**									
Min	0.0	0.0	0.030^‡^	0.0	0.0	0.008^‡^	0.0	0.0	< 0.0001^‡^
Median	35.1	12.3		4.96	0.0		39.1	0.0	
Max	6877.0	2560.4		339.1	370.0		1699.4	35.0	
**Drug amount after cleaning (ng)**									
Min	0.0	0.0	0.018^‡^	0.0	0.0	0.001^‡^	0.0	0.0	0.0002^‡^
Median	3.4	4.6		3.44	0.0		0.0	0.0	
Max	533.3	656.1		695.9	32.8		260.3	0.0	

* Comparison of contamination rates between both Standard and Phaseal groups with a Chi^2^ test

** Comparison of contamination rates between both Standard and Phaseal groups with a Fisher’s exact test

^‡^ Comparison of contamination amounts between both Standard and Phaseal groups with a non-parametric Mann-Whitney test

#### Comparison of contamination between Standard and Phaseal devices after cleaning process

After the cleaning process, there was a decrease in median contamination amounts for the 6 drugs measured (Table 5). However, some high levels of contamination were found for gemcitabine and 5-fluorouracil. A significant difference between the two isolators was evident for cyclophosphamide, ifosfamide and ganciclovir. Ganciclovir was totally absent in the Phaseal isolator after cleaning. For gemcitabine, 5-fluorouracil and cytarabine, no significant difference was observed from one isolator to the other.

### Results of the usability evaluation of devices by pharmacy technicians

**Fig 3:**
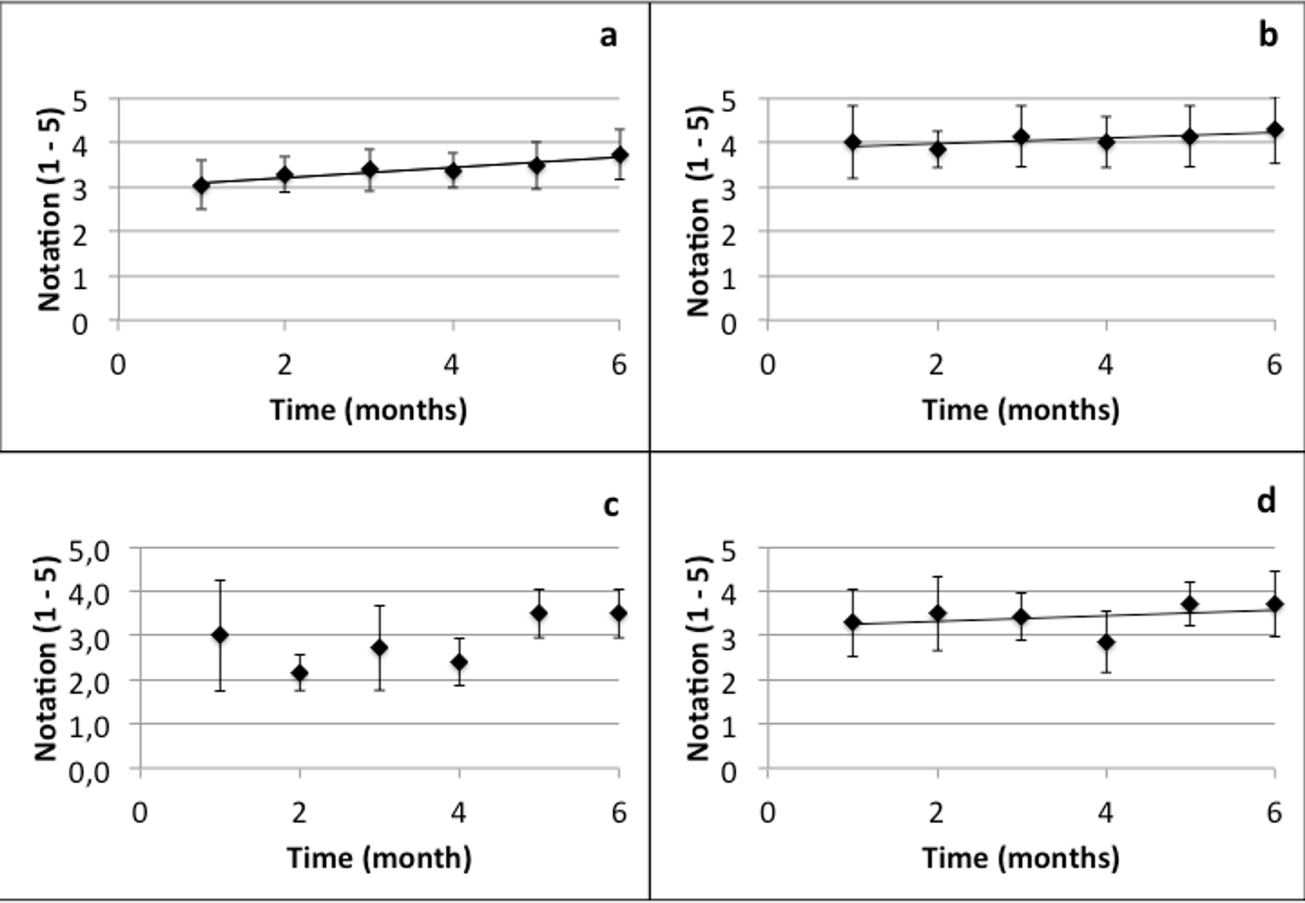
BD-Phaseal assessment throughout the study. Global impression (a), Feeling of safety (b), Encumbrance (c) and Comparison to the reference method (d) are represented. The assessments were made according to a 5-point Likert’s scale. A plot represents the mean and error bars the standard deviation.

Seven pharmacy technicians answered the form each month during the study. The general satisfaction of pharmacy technicians was moderate (medium) at the beginning of the study and increased slightly during the period (M1: 3.04±0.55 *vs*. M6: 3.74±0.56; Fig 3A). Three different items are of particular interest. The feeling of security was good from the start and remained constant throughout the study (Fig 3B). Encumbrance was globally estimated as medium (M1: 3.00±1.26 *vs*. M6: 3.00±0.55). As depicted in Fig 3C, the evolution of this item was biphasic with a decrease between M1 and M4 and an increase at the end of the study. In M4, multi-conditioned protector devices were obtained. These probably helped to change the pharmacy technicians’ perception.

Finally, comparison with the reference method was evaluated as medium at the beginning of the study, but showed a better score towards the end (M1: 3.29±0.76 *vs*. M6: 3.71±0.76). Fig 3B, 3C and 3D show the evolution of these items throughout the study.

## Discussion

Although many medical devices for cytotoxic drug compounding have previously been assessed, few have undergone a comparative and parallel assessment [48]. In the particular case of BD-Phaseal, although numerous studies have previously been published with results in favor of this device [30–45], this study is, to our knowledge, the only prospective, parallel and controlled study comparing this device to standard compounding devices in routine conditions. Several studies aiming to assess this device have been performed in real conditions [21,30,32,36,39–42,45]. Three were performed in new compounding units [30,36] or after renovation [32]. Our results are consistent with those published by Sessink *et al*. in 1999 and Connor *et al*. in 2002 [30,32] but the contamination amounts remain higher than those of Nyman *et al*. [36]. This demonstrates the heterogeneity of occupational exposure to antineoplastic drugs from one unit to another. Apart from the contamination amounts, the main result of our study is that using BD-Phaseal in a newly opened compounding unit leads to a 50% reduction in the number of positive samples compared to standard compounding devices. The effect of the device differs depending on the drug.

However, it should be noted that chemical contamination still exists even when a CSTD is used. A recent study has indeed demonstrated that many pharmacy facilities are contaminated, depending on several factors such as the number of preparations, type of cleaning agent and type of transfer device used [50]. Contamination inside isolators is a multifactorial phenomenon [50,51] because of the contaminated external sides of vials [52], contamination generated during the compounding process, contamination disseminated by hands and the non-optimized decontamination process used. The results observed before the cleaning process are consistent with previously published data [42,45].

Although the device was developed to reduce contamination from antineoplastic drugs inside barrier isolators or laminar airflow hoods and although the team was trained to use the device beforehand to limit technical problems due to human factors, some critical incidents occurred during the study. Thankfully, no vial breakage was recorded in either of the two isolators. Even if the incident rate was low in the “Phaseal” isolator, 15 incidents were recorded with Phaseal devices. Some of them resulted in high contamination. Six of these incidents occurred during the first two weeks of the study. The critical incident which occurred on day 3 involved the breakage of the injector during the injection of gemcitabine solution into the infusion bag and may explain the high accumulated contamination amount for gemcitabine over the 6-month period. Such incidents were classified as incidents due to inadequate knowledge (26.7% of the recorded incidents) or due to procedural errors (73.3% of the recorded incidents). This latter type of incident strongly suggests the necessity to regularly train and educate the team in the use of the device.

The assay developed in the frame of the study has LLOQ comparable to other previous published studies [46,49]. Surprisingly, some drugs were never retrieved (methotrexate, doxorubicine) or very sparsely (dacarbazine, irinotecan). Various factors may account for this such as the incapacity of the wiping method to extract methotrexate and doxorubicin or to detect the presence of very low contamination. According to Nussbaumer et al, wiping with water could limit drug recovery in comparison to a mixture of aqueous solution and organic solvents [46]. In this study, water for injection was chosen because the polymer of the isolator worktop is incompatible with organic solvents. Nevertheless, these drugs were retrieved during the assay development.

According to our results, three groups of drugs may be determined before the cleaning process. For the first group (gemcitabine and fluorouracil), no statistical difference was observed between the two compounding processes. However, gemcitabine contaminated numerous samples and of course the recorded incident in the “Phaseal” isolator increased its mean contamination level. For 5-fluorouracil, even if no significance was reached, contamination was lower in the “Phaseal” isolator. The second group dealt with cytarabine. In this particular case, a significant difference was observed between the compounded doses, which were superior in the “Phaseal” isolator because numerous small doses were prepared for intrathecal injections in the “standard” isolator whereas high doses were prepared for the adult hematological ward in the “Phaseal” isolator. However, although the contamination rate (%) was significantly lower in the “Phaseal” isolator, no statistical difference was observed regarding contamination amount. Finally, the third group involved cyclophosphamide, ifosfamide and ganciclovir. In this group, the same doses were compounded in each isolator. A significant decrease in contamination rate (%) was observed when drugs were compounded with Phaseal. As expected, no significant difference in the compounded doses was noted except for methotrexate and cytarabine and this for the reasons already mentioned above. Our results suggest that the compounded dose is not directly linked to an increase in contamination rate or contamination amount.

As for the pharmacy technicians’ observations, general satisfaction with the device was good from the beginning of the study and increased throughout the study. As our team is aware of the risks of handling cytotoxic drugs, the use of a CSTD was hailed as an additional safety measure. To avoid technical problems due to human factors, the team was trained to handle the device correctly in each compounding situation before the study began. Some points are however worth underlining: firstly, the encumbrance of the worktop is greater than for standard devices. Indeed, at least three devices have to be used for one preparation and the number of Protectors depends on the number of vials. From the fourth month of the study on, we used multi-conditioned devices to reduce encumbrance. This probably helped to modify the pharmacy technicians’ perception (see Fig 3D). Finally, there was an incidence on preparation time, as has previously been demonstrated [53].

Currently, numerous measures are taken to protect handlers from occupational exposure to antineoplastic drugs. Even if no guidelines have as yet been published with clear exposure limits to antineoplastic drugs, the proper use of a CSTD may significantly decrease contamination by these drugs and consequently decrease exposure risk. For this reason our study compares the contamination rates of two different compounding processes by including all positive samples (including traces). Because of the cost of these CSTD devices, the economic aspects should be taken into account but are not included in the present study. Few estimations of the cost/effectiveness balance of such devices have so far been made and would prove useful. In a previous study by Dekyndt *et al*., they were shown to result in a slight increase in preparation cost, as well as in compounding time [54].

Although the study was not designed to assess the effect of the cleaning process, a partial effect of this process on residual contamination was observed. A significant decrease in contamination amount was noted before and after the cleaning process but only for certain drugs. In the particular case of ganciclovir, the use of a CSTD and the standard cleaning procedure with the “Phaseal” isolator resulted in a sharp fall in contamination. This study shows that, even if Phaseal leads to a significant reduction in chemical contamination inside isolators, residual contamination still persists. The cleaning process reduces most of the contamination, but is unable to retrieve all the remaining drugs, owing to their differing chemical structure. An optimized cleaning procedure would probably offer better control of the chemical contamination of barrier isolators [55,56]. This requires further study.

## Conclusion

This prospective study confirms that Phaseal significantly decreases the chemical contamination of barrier isolators compared to standard compounding devices.

## Supporting Information

S1 File Measured contamination according to localization before cleaning process. For both Fig 2 and Table 4, the supporting information consists in the result of the contamination measurement on the different tested localizations. All value are expressed in ng.
(XLS)

S2 File BD-Phaseal assessment throughout the study. The supporting information consists in the assessment of the items by the pharmacy technicians.
(XLS)
